# *QuickStats:* Percentage* of Adults Aged ≥20 Years Consuming Breakfast on a Given Day, by Sex and Age — United States, 2015–2018

**DOI:** 10.15585/mmwr.mm695152a9

**Published:** 2021-01-01

**Figure:**
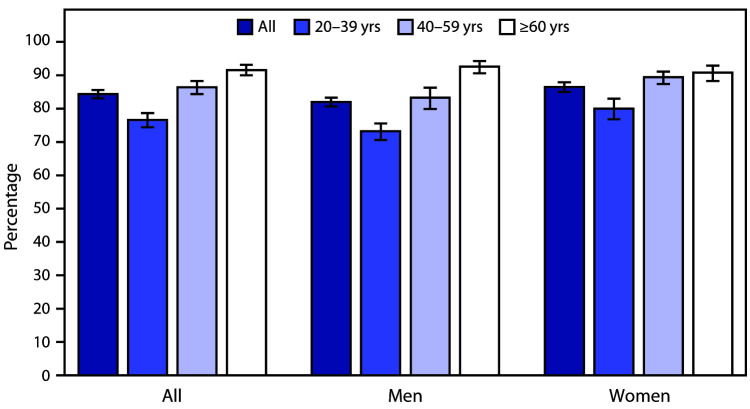
During 2015–2018, 84.4% of adults aged ≥20 years consumed breakfast on a given day, with the percentage increasing with age, from 76.6% among adults aged 20–39 years, to 86.4% among adults aged 40–59 years, and 91.6% among those aged ≥60 years. A higher percentage of women consumed breakfast compared with men among all adults ≥20 years (86.5% versus 82.0%), those aged 20–39 years (80.0% versus 73.2%), and those aged 40–59 years (89.4% versus 83.3%). No significant differences were observed by sex for adults aged ≥60 years (90.8% women and 92.6% men).

**Source:** Terry, AL, Wambogo E. National Health and Nutrition Examination Survey, Dietary Data, 2015–2018; https://www.cdc.gov/nchs/nhanes/index.htm.

